# SEOM-GETTHI clinical guideline for the practical management of molecular platforms (2021)

**DOI:** 10.1007/s12094-022-02817-8

**Published:** 2022-04-01

**Authors:** Juan de la Haba-Rodriguez, Ferran Ferragut Lloret, Maria Angeles Vaz Salgado, Martín Oré Arce, Ana Cardeña Gutiérrez, Jesús García-Donas Jiménez, Carmen Beato Zambrano, Rosa María Rodríguez Alonso, Rafael López López, Nuria Rodriguez Salas

**Affiliations:** 1 Department of Medical Oncology, Hospital Universitario Reina Sofia, Instituto Maimonides de Investigacion Biomedica, Universidad de Córdoba, Córdoba, Spain; 2 Department of Medical Oncology, Hospital General Universitario, Ciudad Real, Spain; 3 Department of Medical Oncology, Hospital Universitario Ramón y Cajal, Madrid, Spain; 4 Department of Medical Oncology, Hospital Marina Baixa de Villajoyosa, Alicante, Spain; 5 Department of Medical Oncology, Hospital Universitario Nuestra Señora de la Candelaria, Tenerife, Spain; 6 Department of Medical Oncology, Centro Integral Oncológico Clara Campal (CIOCC), Madrid, Spain; 7 Department of Medical Oncology, Hospital Universitario de Jerez de la Frontera, Cádiz, Spain; 8 Department of Medical Oncology, Hospital Universitario Reina Sofía, Córdoba, Spain; 9 Department of Medical Oncology, Complejo Hospitalario Universitario de Santiago, La Coruña, Spain; 10 Department of Medical Oncology, Hospital La Paz, P de la Castellana, 261 – 28046 Madrid, Spain

**Keywords:** Molecular platforms, Precision medicine, Next-generation sequencing, Biomarkers

## Abstract

The improvement of molecular alterations in cancer as well as the development of technology has allowed us to bring closer to clinical practice the determination of molecular alterations in the diagnosis and treatment of cancer. The use of multidetermination platforms is spreading in most Spanish hospitals. The objective of these clinical practice guides is to review their usefulness, and establish usage guidelines that guide their incorporation into clinical practice.

## Selecting biomarkers for a molecular platform

In 2011, the Spanish Society of Medical Oncology (SEOM) and the Spanish Society of Pathology launched a joint project to establish guidelines on biomarker testing in patients with advanced NSCLC that have been updated, last time in 2020 as a paradigm, getting the challenge for precision medicine [1].

**Fig. 1:**
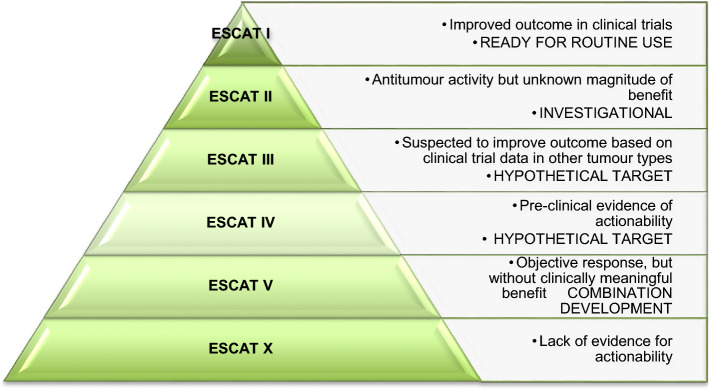
ESMO scale for clinical actionability of molecular targets (ESCAT)

In 2018 the European Society of Medical Oncology (ESMO) defined a scale for clinical actionability of molecular targets in cancer (ESCAT), with the aim of offering a common language to classify genomic alterations based on clinical evidence-based criteria (Fig. 1) [2]. The first recommendation for the use of NGS considering ESCAT was published by ESMO last year. They propose routine use of NGS for advanced non-squamous non-small-cell lung cancer (NSCLC), prostate cancers, ovarian cancers, cholangiocarcinoma and as an alternative to PCR for colorectal (CCR) cancer [3]. In addition, based on the Keynote-158 trial, the tumor mutational burden (TMB) test is also recommended for some tumours: cervical cancers, well and moderately differentiated neuroendocrine tumours, salivary cancers, thyroid cancers, and vulvar cancers [4]. They also encourage clinical research centers to perform it in the context of molecular screening programs to increase the access to innovative drugs and to speed up clinical research.

ASCO guidelines do not include a specific document on recommendations for cancer biomarkers yet. However, they have created the non-free access NCCN Biomarker Compendium^®^ to support decision-making around the use of biomarker tests in cancer patients [5].

We summarize the most relevant biomarkers to develop a personalized and useful NGS platform in oncology practice based on the few recommendations that have been published by scientific societies [6, 7] (Table 1).

**Table 1:** Genetics Biomarkers for precision cancer therapies by tumor type [3–5]

	ESCAT I	ESCAT II	ESCAT III
NSCLC	EGFR 15% del 19, L858R 60% EGFR mutant: acquired T790M exon 20 2–10% uncommon EGFR mutations (exon 18,20,21) 5% ALK 3% MET ex skipping 2% BRAF^v600E^ 1–2% ROS1 0.2–3% NTRK fusions 1–2% RET fusions	3% MET focal amplifications 12% KRAS^G12C^ 2–5% ERBB2	1.2% BRCA 1/2 1.2–7% PI3K 1.7% NRG1
CRC	44% KRAS 4% NRAS 8.5% BRAF^V600E^ 4–5% MSI-H 0.5% NKTR1	2% ERBB2	17% PI3K hotspot mutations 5% ATM mutations 1.7% MET amplifications 1% AKT1^E17K^ 1% TMB-High in MSS 0.3% RET fusions 0.2% ALK fusions
BREAST CANCER	15–20% ERBB2 amplification ER, PR 30–40% PI3K 1% MSI-H 1% NTRK fusions 4% BRCA 1/2 germline mutation androgen receptor and PDL-1 (Triple negative BC)	4% ERBB2 hotspot mutation 3% BRCA 1/2 somatic mutation 10% ESR1(mutation mechanism resistance) 7% PTEN 5% AKT1^E17K^	6% NF1??? 1% MDM2 2% ERBB3
PROSTATE CANCER	9% BRCA1/2 somatic mutations/deletions 1% MSI-H	40% PTEN 5% ATM 1% PALB2	3% PI3K 1% AKT1^E17K^
ENDOMETRIAL CANCER*	2–5% MSI-H, PMS2	ESR1	POLE-aberrant BRAF KRAS PIK3CA PTEN?
OVARIAN*	BRCA 1/2 germline, somatic		ATM, BRIP1, CHEK2, PALB2, RAD51C, RAD 51B
CHOLANGIOCARCINOMA	20% IDH1 mutations 15% FGFR2 mutations 2% MSI-H 2% NKTR fusions	5% BRAF^V600E^ mutations	10% ERBB Amplifications 2% ERBB2 mutations 7% PI3CA hotspot mutations 3% BRCA1/2 mutations 2% MET amplifications
CENTRAL NERVOUS SYSTEM*			1p19q co-deletions IDH1, IDH2 MGMT
SARCOMAS*			MDM2, CDK4 IDH1/IDH2
GIST	KIT, PDGFRA		
PANCREATIC CANCER	1–4% BRCA1/2 germline mutation 1–3% MSI -H < 1% NTRK		3% BRCA 1/2 somatic mutations 90% KRAS mutations 3% PI3CA 3% BRAF^V600E^ 2% MDM2 amplifications 1–2% ERBB2 amplifications/mutations 1% NRG1 fusions < 1% ALK fusions < 1% RET fusions < 1% ROS1 fusions
GASTROESOPHAGEAL ADENOCARCINOMA	16% ERBB2 amplifications 8% MSI-H 2% NTRK fusions	6% EGFR amplifications 3% MET amplifications	3% ERBB2 hotspots mutations 1.3% MET Mutations 7% PI3KCA hotspot mutations 4% FGFR2 amplifications 3% ATM mutations 1–5% BRCA 1/2 mutations < 1% ROS 1 fusions < 1% RET fusions 3% ERBB3 hotspot mutations
MELANOMAS	50% BRAF^v600E^		KIT
HEPATOCELLULAR CARCINOMA	1% NTRK fusions 1% MSI- H		4% PI3CA hotspot mutations 4% MET amplifications 2% RAS mutations

*Non-specific ESCAT available, the classification is proposed regarding SEOM specific tumor type guidelines

Others a site-agnostic biomarkers must be included:

**Neurotrophic receptor tyrosine kinase receptor (NTRK)1–3** incidence is higher in carcinoma of salivary glands (42–100%), secretory breast cancers (90–100%), papillary thyroid carcinoma (2–15%); while they are infrequent, < 1%, in more common adults’ tumours. ESMO recommends using NGS to detect these aberrations only in cancers where this technology is otherwise recommended [8]. SEOM with other societies as Spanish Society of Pathological Anatomy and the Spanish Society of Pediatric Hematology and Oncology has developed a consensus document that includes guidelines on the diagnostic, clinical, and therapeutic aspects of NTRK-fusion tumours proposing NGS for tumours with a high frequency of alterations or in which alterations in NTRK must be known to make a diagnosis [9]. In this scenario, as immunohistochemistry (IHC) is the detection method of choice in most cases, it is necessary to confirm the fusion of the NTRK genes by NGS before initiating NTRK inhibitors, which has shown promising activity in early phase pan-tumor trials [10, 11].

**PD1 and PD-L1 status**, assessed by IHC staining, has been identified as a biomarker associated with a higher chance of tumor response in patients treated with anti-PD-L1 antibodies and a better OS in multiple tumor types [12].

**TMB high** have been correlated with overall survival benefits following treatment with ipilimumab in melanoma, pembrolizumab in NSCLC and atezolizumab in bladder cancers. It is currently believed that a high TMB yields numerous immunogenic cancer cell neo-epitopes that may be recognized by T cells upon presentation by MHC molecules. However, TMB seems to be a prognostic marker independent of the intratumorally inflammatory gene expression profile [12].

**Mismatch repair status**. Tumours with DNA mismatch repair deficiency (dMMR) have shown great sensitivity to anti-PD-L1 therapies. It is currently believed that tumours harboring an erroneous MMR system will accumulate DNA mutations, which can lead to the presence of high levels of mutation-associated neoantigens (MSI-H), most recognized by immune cells. dMMR/MSI-H status is an approved biomarker for pembrolizumab [12].

**Some mutations ca be germline and or somatic.** Germline alterations require confirmation in matched normal samples from the tumor-bearing host and after confirming is also necessary to perform cascade testing on family members. Table 2 [13].

**Table 2:** Somatic mutations that should be referred to genetic counseling [15]

Germline associated syndrome	Mutation	Main cancers involved
Li-Fraumeni	TP53	Sarcomas, breast, and brain
Lynch	MSH2, MLH1, MSH6, PMS2	Gastrointestinal tract, endometrium, ovary, brain, breast, renal pelvis
Hereditary breast-ovarian cancer	BRCA1, BRCA2	Breast, ovary, prostate, pancreas
Familial adenomatous polyposis	APC, MUTYH	CCR, small bowel, stomach, brain, bone, skin
Hereditary diffuse gastric cancer	CDH1	Stomach, breast
Familial atypical multiple melanoma	CDK4, CDKN2A	Melanoma, pancreatic cancer, astrocytoma
Werner	MEN1	Pancreatic, pituitary gland tumors
Retinoblastoma	RB1	Eye, pineal gland, osteosarcoma, melanoma, soft tissue sarcoma
Multiple endocrine neoplasia type 2	RET	Medullary thyroid cancer, pheochromocytoma
Von Hippel-Lindau	VHL	Kidney
Peutz-Jeghers	STK11	Breast, CCR, pancreas, stomach, hamartomas
Familial paraganglioma	SDHD, SDHB, SDHC	Paragangliomas, pheochromocytomas
Bir-Hoge-Dube	FLCN	Chromophobe renal cell cancer
Tuberous sclerosis	TSC1/2	Angiofibroma, angiomyolipoma, giant cell astrocytoma
Neurofibromatosis type 1	NF1	Optic gliomas, neurofibromas
Neurofibromatosis type 2	NF2	Schwannomas, meningiomas, gliomas, neurofibromas
Gorlin	PTCH1	Childhood primitive neuroectodermal tumors, skin basal cell carcinomas
Juvenile polyposis	BMPR1A, SMAD4	Multiple non-cancerous growth in the colon

## Genetics platforms for hereditary cancer syndromes

Genetic diagnosis of hereditary cancer syndromes offers the opportunity to establish more effective predictive and preventive measures for the patient and their families. Next Generation Sequencing (NGS) technologies have transformed hereditary cancer syndromes testing process. Several multigene panels (MP) offers an important improvement in the efficiency of genetic diagnosis, but there is a debate about what genes should and should not be tested because of lack of actionability. Multi-gene testing may be most useful when more than one gene can explain an inherited cancer syndrome [14]. Although clinical criteria for genetic testing continue to be largely based on personal and family history with around a 10% detection rate, broader criteria are being applied with a lower threshold for detecting mutations when there are therapeutic implications for patients [15]. Assess for germline BRCA1/2 mutations in all patients with recurrent or metastatic breast cancer to identify candidates for PARP inhibitor therapy biomarkers for platinum therapy [16] and PARP inhibitors [17]. Another therapeutically actionable germline variants are in *CHEK2,* ATM*,* mismatch repair genes (MLH1, MSH2, MSH6, PMS2, and EPCAM), and PALB2. Patients with microsatellite instability treatment with checkpoint inhibitors therapy can be considered when is available [18].

There are several issues to consider regarding multi-gene testing. Commercially available tests may differ significantly on a number of factors, such as number of genes analyzed among others. Therefore, the specific laboratory and multi-gene test should be chosen carefully [14] (syndrome-specific panel, cancer-specific panel, multi cancer panel, etc.).Genetic counselling by clinicians with specific training or expertise should always be offered before ordering germinal testing.

The presence of a BRCA1 or BRCA2 mutation accounts for the majority of hereditary breast and ovarian cancer syndromes. BRACAnalysis identifies patients with BRCA 1/2 mutation and allows therapy personalized. Genetic susceptibility to breast or ovarian cancer might also be associated with mutations in other high and moderate penetrance genes, some of which are associated with known hereditary cancer syndromes, such as p53, PTEN, NF1, CDH1, STK11, MLH1, MSH2, MSH6 and PMS2, BARD1, PALB2, CHEK2, ATM, RAD51C, RAD51D and BRIP1.Therefore, HBOC germline panels including these genes are recommended (II, A) [19, 20].

Germline mutations commonly found in pancreatic adenocarcinoma include BRCA1, BRCA2, CDKN2A, mismatch repair genes associated with Lynch syndrome, ATM, PALB2, STK11, and TP53 [21–23]. In addition, hereditary pancreatitis, which is associated with a significantly increased risk for pancreatic cancer, is associated with the genes PRSS1 and SPINK1 [24, 25].

Consider cancer predisposition next-generation sequencing (NGS) panel testing, which includes BRCA1, BRCA2, ATM, PALB2, CHEK2, MLH1, MSH2, MSH6, and PMS2 in patients with prostate cancer who meets criteria [III, A] [26].

Genetic susceptibility to CRC includes well defined inherited syndromes such as Lynch syndrome, familial adenomatous polyposis (FAP), and MutY human homolog (MUTYH)-associated polyposis (MAP). Other entities include Muir-Torre, Turcot, Gardner, Cowden, Bannayan-Riley-Ruvalcaba, Peutz-Jeghers, juvenile polyposis, and serrated polyposis syndromes [27].

Syndrome specific testing of the panel of genes that cause Lynch syndrome (MLH1, MSH2, MSH6, PMS2, and EPCAM) may be considered for individuals who meet criteria for Lynch Syndrome [28].

Patients with multiple colorectal adenomas (> 10) should be considered for panel germline genetic testing that includes APC, MUTYH, POLE, POLD1, GREM1 and NTHL1 genes [III, A] [29–31].

## Genetics platforms for unknown primary tumours

At this time of medicine based on precision, one of the challenges of oncology is to diagnose the origin of the tumor to direct the treatment with greater precision and enhance the therapeutic results in tumours with low sensibility to the treatment with chemotherapy [32]. In 3–5% of malignant neoplasms, the primary origin is unknown, which is a challenge when it is time to select a treatment. The use of traditional diagnostic procedures makes it easier to identify the tissue of origin in 30%[33]. The predictive informed precisions are about 80–90% [34, 35] with the use of current molecular platforms.

Different molecular diagnostic platforms evaluate the genomic expression, identify the tumor through a classifier of the type of cancer based on firms of DNA methylation microarrays or a RNA classifier based on tumor samples [35–37]. The molecular similarity of the tumor sample is quantified with a reference database with the tumors selected by the different platforms. There are different trading platforms (Table 3).

**Table 3:** Main trading platforms for unknown origin neoplasm [37–46]

Plataform	Method	No. of genes	Sensitivity (%)
Ques-Lab	RT-PCR	92	–
Veridex	RT-PCR	6	76.0
Pathwork	cDNA array	2000	89.0
Cup-Print	cDNA array	495	85.0
Rossetta	miRNA array	64	90.0
CancerType	RT-PCR	92	89.0
EPICUP	Methylation array	–	97.7
CUP-AI-Dx	ARN-seq	Transcriptional profiles of 18,217 tumors	EEUU: 86.96% Australia: 72.46%

One of the main limits of molecular platforms is to have enough quantity, quality and percentage of tumor cells. The diagnostic use with immunohistochemistry through the use of antibodies directed to protein antigens is a standardized test that can exhaust the tumor tissue for its use in future diagnostic procedures, although it correlates well with the platforms. Diagnosis is sometimes difficult in orphan tumors and not very frequent or with unusual histopathology features because they are not included in the non-included features in the database of the platforms (Table 4).

**Table 4:** Recommendations and evidences

In daily practice, we are encouraged to obtain a customized NGS platform that includes ESCAT level I gene alterations	I-II/B
Clinical oncologist´s education about when and how to interpret molecular maps is essential to benefit patients from modern approaches through tumor profiling	V/C
Use of liquid biopsy to achieve precision oncology, especially in rare tumors	II/B

Another limit of these platforms is that they are not subsidized by the National Health System. In the economic analysis, EPICUP showed profitable in breast, colon, pancreas, lung (NSCLC), hepatocellular and prostate cancers in comparison with other available alternatives, increasing the amount of well-treated patients, directing the therapy and with a cost-effectiveness benefit [47].

The use of molecular diagnostic platforms helps to direct treatments in tumors with a low sensibility towards chemotherapy and also offers a very useful tool for the diagnosis of tumors with an unknown origin.

## Predictive platforms

Molecular platforms may be useful tools to replace some clinically available IHQ, FISH and RT-PCR assays as the initial molecular diagnostic due to its cost effectiveness, and also, to be a tissue-saving option [48]. That said, we will proceed to review the currently approved molecular platforms that could be useful to predict response to target therapy for metastatic disease. No molecular platforms are approved yet for localized disease during daily clinical practice [49].

### Colorectal cancer (CRC)

*Praxis Extended RAS Panel* is a NGS based in vitro diagnostic for evaluating 56 KRAS/NRAS mutations to determine patient’s eligibility for treatment with EGFR monoclonal antibodies (mAbs) [50]. Testing KRAS/BRAF is only recommended for stage IV CRC, and consequently, molecular platforms are not approved yet for other stages [51]. MMR or MSI testing is universally approved for all stages. Stage II MSI-H patients may have a good prognosis and do not benefit from 5-FU adjuvant therapy. Also, they do benefit from anti PD-L1 treatment for advanced disease [52].

### BRCA status to predict response to PARP inhibitors

Germline BRCA1 and BRCA2 status is a critical biomarker to help determine the appropriate therapy for patients with ovarian, prostate, pancreatic and breast tumors [53]. *BRACAnalysis CDx* detects germline mutations only, not somatic mutations from a patient’s blood sample [54]. *FoundationFocusTM CDxBRCA* and *Myriad myChoice* CDx are *NGS* based in vitro diagnostic device for qualitative detection of *BRCA1* and *BRCA2* alterations in formalin-fixed paraffin-embedded (FFPE) ovarian tumor tissue. These test does not provide information about susceptibility [55, 56]. Furthermore, *Myriad myChoice* CDx determinates Genomic Instability Score (GIS) using DNA isolated from FFPE tumor tissue specimens.

### Non-small-cell lung cancer (NSCLC)

Molecular platforms can be extremely useful for non-squamous NSCLC, the solid tumor with the widest variety of potential therapeutic targets [57]. *Oncomine Dx Target Test*: Detects 46 cancer driver gene variants, including EGFR mutations (including L858R, T790M, and exon 19 deletions); BRAF, KRAS, ERBB2, and MET exon 14 skipping mutations; and ALK, ROS1, RET, and NTRK1/2/3 fusions [58].

### Solid tumors

*FoundationOne* CDx was the first FDA-approved tissue-based broad companion diagnostic (CDx) that is clinically and analytically validated for all solid tumors. Test results include MSI, TMB and loss of heterozygosity (LOH) for ovarian cancer patients [59]. *Memorial Sloan Kettering-Integrated Mutation Profiling of Actionable Cancer Targets (MSK-IMPACT)* [60]. A hybridization capture-based NGS assay for targeted deep sequencing of all exons and selected introns of 341 key cancer genes in FFPE tumors. *Omics Core and PGDx elio*™ tissue complete use NGS of FFPE tumor tissue to detect both TMB and some information about point mutations and small insertions and deletions. PGDx also analyzes MSI status.

## Prognostic platforms

Predicting the risk of recurrence is critical to optimize adjuvant treatment. Diverse gene-based assays may be used to gain additional prognostic and/or predictive information to complement pathology assessment [61]. Breast and Colon cancer has the developed platforms:

For early breast cancer Oncotype, Mammaprint, Nanostring are useful are useful for estimating the risk of recurrence and the benefit of adjuvant chemotherapy treatment in patients without metastatic axillary nodal involvement. Recently Oncotype has also shown usefulness for postmenopausal patients with nodal involvement [62–65].

The use of adjuvant CT in patients with stage II colorectal cancer (CRC) is controversial. Multigenic tests have been developed to identify patients with higher risk of recurrence, who may benefit more from adjuvant CT. However, clinicians and patients may consider their use to complement clinicopathological information. Only Oncotype DX [66] and GeneFx Colon [67] have been validated in stage II CRC. Their use might be considered on intermediate-risk stage II scenarios: i.e. to treat T3 N0 classified as high risk by the signature, or for avoiding chemotherapy in T4 N0 classified as low risk by the signature. Immunoscore has been validated in patients with stage I-III CRC [68]. It could be considered to stablish the prognosis of patients used in conjunction with the TNM scoring and thus support the CT decision-making process in stage II and even in low-risk stage III patients.

## Liquid biopsy

The term liquid biopsy was first described by K. Pantel and C. Alix-Pambieres to study circulating tumor cells (CTCs) in the blood of cancer patient [69]. Currently it has been expanded to study circulating tumor nucleic acids (DNA and RNA) as well as other structures such as exosomes and platelets, extending to all biological fluids such as urine, cerebrospinal fluid, and others. The popularization of the liquid biopsy is due to the convenience and reproducibility to explore the alterations in the circulating free DNA. This approach is the current and future development field of oncology.

The initial concerns about the correlation with the titular biopsy has almost been overcome, the complementary or even principal information provided by liquid biopsy to the management of solid tumors seems superior, although it needs to demonstrate its clinical utility in most clinical situations like treatment selection, disease monitoring, minimal residual disease study and anticipating resistance, and finally early diagnosis [70].

In practice, there are two principal methods for studying ctDNA in plasma, based on PCR and by next generation sequencing (NGS). PCR-based detection of cfDNA, especially with the new digital PCR systems, is highly sensitive and easy to interpret with the limitation that can be studied only a few previously known mutations. The most promising development is with the NGS panels that study several genes at the same time or de complete genome, but where the sensitivity is lower, although it is improving and requires complex equipment and a very high bio-informatics support. Intriguingly, the solitary publication of liquid biopsy and rare tumors is a clinical case of a hemangiopericytoma where try to characterize the CTCs [71].

Current evidence is that genotyping of cfDNA in plasma can be complementary to the tissue and vice versa, in patients with advanced disease to identify a biomarker for initiate targeted treatment [72]. Currently there are some tests to study ctDNA that are approved by FDA as companion diagnostics in some cancers and for some targeted treatments, although nonspecific for rare tumors that were underrepresented in the studies.

## Final considerations

Currently, there are three major barriers for a wide implementation of precision oncology: restrictions in the access to molecular platforms, availability of targeted drugs for transversal indications and physicians skills in the interpretation of molecular results [73, 74]. Though universal testing should become a reality in the near future, doctors must decide what patients are more likely to benefit from a molecular platform, select the most appropriate tool and grant access to therapy in case of a “positive” finding [75].

The changing landscape of personalized medicine, where not only new drugs but also new platforms become available quickly, makes this decision particularly challenging. Additionally, cancer has shown a biological plasticity that leads to the appearance of new alterations along the course of the disease [76]. Thus, molecular testing should be repeated in cases where the understanding of the mechanism of resistance could lead to alternative therapeutic options (for instance EGFR mutations in lung cancer) [77].

As a result, oncologists do not only need to decide who must be tested but also how many times and when. A new and dynamic approach to this situation should replace the traditional model where guidelines or recommendations are fixed regularly.

Molecular Tumor Boards (MTB) have emerged as the best way to support clinicians struggling with precision medicine in daily practice. These committees should include genetic counselors and biologists able to interpret the results of molecular platforms, oncologists specialized in different areas (since drugs approved in one tumor could be interesting in a different indication) and personal from clinical trials units to ensure a wide access to targeted therapies [78].

Since many institutions cannot grant such multidisciplinary environment, initiatives like the GETTHI National Molecular Tumor Board, where any oncologist can submit a case for consideration (https://www.gethi.org/contenidos/investigacion/tumorBoard.aspx), could help to ensure that every patient gets the most updated management.

## References

[1] Rodríguez- Lescure A, de la Peña FA, Aranda E (2020). Study of the Spanish Society of Medical Oncology (SEOM) on the access to oncology drugs and predictive biomarkers in Spain. Clin Trans Oncol.

[2] Mateo J, Chakravarty D, Dienstmann R (2018). A framework to rank genomic alterations as targets for cancer precision medicine: the ESMO Scale for Clinical Actionability of molecular Targets (ESCAT). Anns Oncol.

[3] Mosele F, Remon J, Westphalen CB (2020). Recommendations for the use of next-generation sequencing (NGS) for patients with metastatic cancers: a report from the ESMO Precision Medicine Working Group. Anns Oncol.

[4] Marabelle A, Marwan F, López J (2020). Association of tumour mutational burden with outcomes in patients with advanced solid tumors treated with pembrolizumab: prospective biomarker analysis of the multicohort, open-label, phase 2 Keynote-158 study. Lancet Oncol.

[5] National Comprehensive Cancer Network Biomarkers Compendium 2021. https://www.nccn.org/compendia-templates/compendia/biomarkers-compendium

[6] Garrido P, Aldaz A, Vera R (2018). Proposal for the creation of a national strategy for precision medicine in cancer: a position statement of SEOM, SEAP and SEFH. Clin Trans Oncol.

[7] Garrido P, Conde E, de Castro J (2020). Updated guidelines for predictive biomarker testing in advanced non-small-cell lung cancer: a National Consensus of the Spanish Society of Pathology and the Spanish Society of Medical Oncology. Clin Trans Oncol.

[8] Marchio C, Scaltri M, Landanyl M (2019). ESMO recommendations on the standard methods to detect NTRK fusions in daily practice and clinical research. Anns Oncol.

[9] Garrido P, Hladun R, de Álava R (2021). Multidisciplinary consensus on optimising the detection of NKTR gene alterations in tumours. Clin Trans Oncol.

[10] Hong DS, DuBois SG, Kummar S (2020). Larotrectinib in patients with TRK fusion-positive solid tumours: a pooled analysis of three phase 1/2 clinical trials. Lancet Oncol.

[11] Doebel RC, Drilon A, Paz-Ares L (2020). Entrectinib in patients with advanced or metastatic NTRK fusion-positive solid tumours: integrated analysis of three phase 1–2 trials. Lancet Oncol.

[12] ESMO Handbook of therapeutics biomarkers in Oncology

[13] Sección SEOM Cáncer Familiar y Hereditario. Cáncer Hereditario. 3ª edición. Sociedad Española de Oncología Médica, 2019. ISBN: 978-84-09-10462-8

[14] Hall MJ, Forman AD, Pilarski R (2014). Gene panel testing for inherited cancer risk. J Natl Compr Canc Netw.

[15] González-Santiago S, Ramón y Cajal T, Aguirre E (2020). SEOM clinical guidelines in hereditary breast and ovarian cancer (2019). Clin Transl Oncol.

[16] Cheng HH, Pritchard CC, Boyd T (2016). Biallelic inactivation of BRCA2 in platinum-sensitive metastatic castration-resistant prostate cancer. Eur Urol.

[17] Mateo J, Carreira S, Sandhu S (2015). DNA-repair defects and olaparib in metastatic prostate cancer. N Engl J Med.

[18] Le DT, Durham JN, Smith KN (2017). Mismatch repair deficiency predicts response of solid tumors to PD-1 blockade. Science.

[19] Feliubadaló L, López-Fernández A, Pineda M, Díez O, Del Valle J, Gutiérrez-Enríquez S (2019). Opportunistic testing of BRCA1, BRCA2 and mismatch repair genes improves the yield of phenotype driven hereditary cancer gene panels. Int J Cancer.

[20] Manchanda R, Patel S, Gordeev VS (2018). Cost-effectiveness of population-based BRCA1, BRCA2, RAD51C, RAD51D, BRIP1, PALB2 mutation testing in unselected general population women. J Natl Cancer Inst.

[21] Holter S, Borgida A, Dodd A (2015). Germline BRCA mutations in a large clinic-based cohort of patients with pancreatic adenocarcinoma. J Clin Oncol.

[22] Salo-Mullen EE, O'Reilly EM, Kelsen DP (2015). Identification of germline genetic mutations in patients with pancreatic cancer. Cancer.

[23] Hu C, Hart SN, Polley EC (2018). Association between inherited germline mutations in cancer predisposition genes and risk of pancreatic cancer. JAMA.

[24] Grant RC, Selander I, Connor AA (2015). Prevalence of germline mutations in cancer predisposition genes in patients with pancreatic cancer. Gastroenterology.

[25] Petersen GM (2016). Familial pancreatic cancer. Semin Oncol.

[26] Nicolosi P, Ledet E, Yang S (2019). Prevalence of germline variants in prostate cancer and implications for current genetic testing guidelines. JAMA Oncol.

[27] Burt R, Neklason DW (2005). Genetic testing for inherited colon cancer. Gastroenterology.

[28] Soares BL, Brant AC, Gomes R (2018). Screening for germline mutations in mismatch repair genes in patients with Lynch syndrome by next generation sequencing. Fam Cancer.

[29] Stjepanovic N, Moreira L, Carneiro F (2019). Hereditary gastrointestinal cancers: ESMO Clinical Practice Guidelines for diagnosis, treatment and follow-up. Ann Oncol.

[30] Palles C, Cazier JB, Howarth KM (2013). Germline mutations affecting the proofreading domains of POLE and POLD1 predispose to colorectal adenomas and carcinomas. Nat Genet.

[31] Rohlin A, Eiengard F, Lundstam U (2016). GREM1 and POLE variants in hereditary colorectal cancer syndromes. Genes Chromosomes Cancer.

[32] Fizazi K, Maillard A, Penel N (2019). A phase III trial of empiric chemotherapy with cisplatin and gemcitabine or systemic treatment tailored by molecular gene expression analysis in patients with carcinomas of an unknown primary (CUP) site (GEFCAPI 04). Ann Oncol.

[33] Moran S, Martinez-Cardús A, Boussios S (2017). Precision medicine based on epigenomics: the paradigm of carcinoma of unknown primary. Nat Rev Clin Oncol.

[34] Monzon FA, Lyons-Weiler M, Buturovic LJ, Rigl CT, Henner WD, Sciulli C, Dumur CI, Medeiros F, Anderson GG (2009). Multicenter validation of a 1550-gene expression profile for identification of tumor tissue of origin. J Clin Oncol.

[35] Rosenfeld N, Aharonov R, Meiri E (2008). MicroRNAs accurately identify cancer tissue origin. Nature Biotechnol.

[36] Moran S, Martínez-Cardús A, Sayols S, Musulén E, Balañá C, Estival-Gonzalez A, Moutinho C, Heyn H, Diaz-Lagares A, de Moura MC, Stella GM, Comoglio PM, Ruiz-Miró M, Matias-Guiu X, Pazo-Cid R, Antón A, Lopez-Lopez R, Soler G, Longo F, Guerra I, Fernandez S, Assenov Y, Plass C, Morales R, Carles J, Bowtell D, Mileshkin L, Sia D, Tothill R, Tabernero J, Llovet JM, Esteller M (2016). Epigenetic profiling to classify cancer of unknown primary: a multicentre, retrospective analysis. Lancet Oncol.

[37] Zhao Y, Pan Z, Namburi S (2020). CUP-AI-Dx: a tool for inferring cancer tissue of origin and molecular subtype using RNA gene-expression data and artificial intelligence. EBioMedicine.

[38] Erlander MG, Ma XJ, Kesty NC, Bao L, Salunga R, Schnabel CA (2011). Performance and clinical evaluation of the 92-gene real-time PCR assay for tumor classification. J Mol Diagn.

[39] Kerr SE, Schnabel CA, Sullivan PS, Zhang Y, Singh V, Carey B (2012). Multisite validation study to determine performance characteristics of a 92-gene molecular cancer classifier. Clin Cancer Res.

[40] Wu F, Huang D, Wang L, Xu Q, Liu F, Ye X (2012). 92-Gene molecular profiling in identification of cancer origin: a retrospective study in Chinese population and performance within different subgroups. PLoS ONE.

[41] Grewal JK, Tessier-Cloutier B, Jones M, Gakkhar S, Ma Y, Moore R (2019). Application of a neural network whole transcriptome-based pan-cancer method for diagnosis of primary and metastatic cancers. JAMA Netw Open.

[42] Tothill RW, Shi F, Paiman L, Bedo J, Kowalczyk A, Mileshkin L (2015). Development and validation of a gene expression tumour classifier for cancer of unknown primary. Pathology.

[43] Penson A, Camacho N, Zheng Y, Varghese AM, Al-Ahmadie H, Razavi P (2019). Development of genome-derived tumor type prediction to inform clinical cancer care. JAMA Oncol.

[44] Pillai R, Deeter R, Rigl CT, Nystrom JS, Miller MH, Buturovic L (2011). Validation and reproducibility of a microarray-based gene expression test for tumor identification in formalin-fixed, paraffin-embedded specimens. J Mol Diagn.

[45] Meiri E, Mueller WC, Rosenwald S, Zepeniuk M, Klinke E, Edmonston TB (2012). A second-generation microRNA-based assay for diagnosing tumor tissue origin. Oncologist.

[46] Losa F, Iglesias L, Pané M, Sanz J, Nieto B, Fusté V, de la Cruz-Merino L, Concha Á, Balañá C, Matías-Guiu X (2018). Consensus statement by the Spanish Society of Pathology and the Spanish Society of Medical Oncology on the diagnosis and treatment of cancer of unknown primary. Clin Transl Oncol.

[47] Balana C, Gracia A, Kaskens L, Chiavenna S, Matias-Guiu X, Rubio-Rodriguez D, Rubio-Terres C, Iglesias L, Esteller M (2015). Economic analysis of EPICUP, an epigenetic test to predict the tissue of origin in cancer of unknown primary site. J Clin Oncol.

[48] Chang KTE, Goytain A, Tucker T, Karsan A, Lee CH, Nielsen TO (2018). Development and evaluation of a pan-sarcoma fusion gene detection assay using the nanostring ncounter platform. J Mol Diagnostics [Internet]..

[49] Mosele F, Remon J, Mateo J, Westphalen CB, Barlesi F, Lolkema MP (2020). Recommendations for the use of next-generation sequencing (NGS) for patients with metastatic cancers: a report from the ESMO Precision Medicine Working Group. Ann Oncol [Internet]..

[50] Udar N, Iyer A, Porter M, Haigis R, Smith S, Dhillon S (2020). Development and analytical validation of a DNA dual-strand approach for the US Food and Drug Administration-approved next-generation sequencing-based praxis extended RAS panel for metastatic colorectal cancer samples. J Mol Diagnostics [Internet]..

[51] García-Alfonso P, García-Carbonero R, García-Foncillas J, Pérez-Segura P, Salazar R, Vera R (2020). Update of the recommendations for the determination of biomarkers in colorectal carcinoma: National Consensus of the Spanish Society of Medical Oncology and the Spanish Society of Pathology. Clin Transl Oncol [Internet]..

[52] Ciardiello D, Vitiello PP, Cardone C, Martini G, Troiani T, Martinelli E (2019). Immunotherapy of colorectal cancer: challenges for therapeutic efficacy. Cancer Treat Rev [Internet]..

[53] Rodríguez N, Viñal D, Rodríguez-Cobos J, De Castro J, Domínguez G (2020). Genomic profiling in oncology clinical practice. Clin Transl Oncol [Internet]..

[54] Gunderson CC, Moore KN (2015). BRACAnalysis CDx as a companion diagnostic tool for Lynparza. Expert Rev Mol Diagn [Internet]..

[55] Ford L, Wolford JE, Brown SM, Randall LM (2020). A profile on the FoundationFocus CDxBRCA tests. Expert Rev Mol Diagn [Internet]..

[56] Washington CR, Moore KN (2021). PARP inhibitors in the treatment of ovarian cancer. Curr Opin Obstet Gynecol.

[57] Garrido P, Conde E, de Castro J, Gómez-Román JJ, Felip E, Pijuan L (2020). Updated guidelines for predictive biomarker testing in advanced non-small-cell lung cancer: a National Consensus of the Spanish Society of Pathology and the Spanish Society of Medical Oncology. Clin Transl Oncol [Internet]..

[58] Ariyasu R, Uchibori K, Ninomiya H, Ogusu S, Tsugitomi R, Manabe R (2021). Feasibility of next-generation sequencing test for patients with advanced NSCLC in clinical practice. Thorac Cancer [Internet]..

[59] Woodhouse R, Li M, Hughes J, Delfosse D, Skoletsky J, Ma P (2020). Clinical and analytical validation of FoundationOne Liquid CDx, a novel 324-Gene cfDNA-based comprehensive genomic profiling assay for cancers of solid tumor origin. PLoS One [Internet]..

[60] Cheng DT, Mitchell TN, Zehir A, Shah RH, Benayed R, Syed A (2015). Memorial Sloan Kettering-Integrated mutation profiling of actionable cancer targets (MSK-IMPACT): a hybridization capture-based next-generation sequencing clinical assay for solid tumor molecular oncology. J Mol Diagn [Internet]..

[61] Colomer R, Aranda-López I, Albanell J (2018). Biomarkers in breast cancer: a consensus statement by the Spanish Society of Medical Oncology and the Spanish Society of Pathology [published correction appears in Clin Transl Oncol. Jun 2018]. Clin Transl Oncol.

[62] Paik S, Tang G, Shak S (2006). Gene expression and benefit of chemotherapy in women with node-negative, estrogen receptor-positive breast cancer. J Clin Oncol.

[63] Sparano JA, Gray RJ, Makower DF (2018). Adjuvant chemotherapy guided by a 21-gene expression assay in breast cancer. N Engl J Med.

[64] Kalinsky K, Barlow WE, Meric-Bernstam F et al. SWOG S1007: Adjuvant trial randomized ER+ patients who had a recurrence score <25 and 1–3 positive nodes to endocrine therapy (ET) versus ET + chemotherapy. Presented at the 2020 San Antonio Breast Cancer Symposium (SABCS). December 8–11, 2020. Abstract GS3-01

[65] van de Vijver MJ, He YD, van’t Veer LJ (2002). A gene expression signature as a predictor of survival in breast cancer. N Engl J Med.

[66] Cardoso F, van’t Veer LJ, Bogaerts J (2016). 70-gene signature as an aid to treatment decisions in early-stage breast cancer. N Engl J Med.

[67] Gray RG, Quirke P, Handley K (2011). Validation study of a quantitative multigene reverse transcriptase-polymerase chain reaction assay for assessment of recurrence risk in patients with stage II colon cancer. J Clin Oncol.

[68] Pagès F, Mlecnik B, Marliot F (2018). International validation of the consensus Immunoscore for the classification of colon cancer: a prognostic and accuracy study. Lancet.

[69] Pantel K, Alix-Panabieres C (2010). Circulating tumour cells in cancer patients: challenges and perspectives. Trends Mol Med.

[70] Ignatiadis M, Sledge GW, Jeffrey SS (2021). Liquid biopsy enters the clinic—implementation issues and future challenges. Nat Rev Clin Oncol.

[71] Nicolazzo C, Colangelo L, Corsi A (2018). Liquid biopsy in rare cancers: lessons from hemangiopericytoma. Anal Cell Pathol.

[72] Remon J, García-Campelo R, de Álava E (2020). Liquid biopsy in oncology: a consensus statement of the Spanish Society of Pathology and the Spanish Society of Medical Oncology. Clin Transl Oncol.

[73] Levit LA, Kim ES, McAneny BL, Nadauld LD, Levit K, Schenkel C, Schilsky RL (2019). Implementing precision medicine in community-based oncology programs: three models. J Oncol Pract.

[74] Yam C, Ma BBY, Yap TA (2021). Global implementation of precision oncology. JCO Precis Oncol.

[75] Singla P, Musyuni P, Aggarwal G, Singh H (2021). Precision medicine: an emerging paradigm for improved diagnosis and safe therapy in pediatric oncology. Cureus.

[76] Meacham CE, Morrison SJ (2013). Tumour heterogeneity and cancer cell plasticity. Nature.

[77] Yasuda H, Kobayashi S, Costa DB (2012). EGFR exon 20 insertion mutations in non-small-cell lung cancer: preclinical data and clinical implications. Lancet Oncol.

[78] Delman KA (2020). Introducing the "virtual tumor board" series in CA: a cancer journal for clinicians. CA Cancer J Clin.

